# Outcome of flat bone sarcomas (other than Ewing's) in children and adolescents: a study of 25 cases

**DOI:** 10.1038/sj.bjc.6601564

**Published:** 2004-02-03

**Authors:** V Minard-Colin, C Kalifa, J-M Guinebretiere, L Brugieres, J Dubousset, J-L Habrand, G Vassal, O Hartmann

**Affiliations:** 1 Department of Pediatrics, Institut Gustave Roussy, 39 rue Camille Desmoulins, Villejuif Cedex 94805, France; 2 Department of Pathology, Institut Gustave Roussy, 39 rue Camille Desmoulins, Villejuif Cedex 94805, France; 3 Department of Radiotherapy, Institut Gustave Roussy, 39 rue Camille Desmoulins, Villejuif Cedex 94805, France

**Keywords:** flat bone sarcoma, osteosarcoma, children, adolescents, prognostic factors

## Abstract

We analysed the clinical features and outcome of young patients with non-Ewing's flat bone sarcoma treated during the era of contemporary chemotherapy. The characteristics and outcome of 25 patients (15 males and 10 females) with primary or radiation-related flat bone sarcoma treated in the Pediatrics Department at the Institut Gustave Roussy from 1981 to 1999 were reviewed. In all, 20 patients had osteosarcoma, four chondrosarcoma and one malignant fibrous histiocytoma. The age at diagnosis ranged from 2 to 23 years (median, 15 years). Nine tumours were located in the craniofacial bones, 11 in the pelvis and five in flat bones at other sites. Four patients had metastatic disease at diagnosis. Radiation-associated flat bone osteosarcoma was diagnosed in 10 out of 25 cases. The projected overall survival and event-free survival (EFS) rates at 5 years were 45.1 and 34.3% for all the 25 patients. The EFS rate of patients with second bone sarcoma was similar to that of patients with *de novo* flat bone sarcoma (*P*=0.1). The aim of treatment was curative for 24 patients, 23 of whom were treated with intensive chemotherapy regimens and 19 with surgery. Significant adverse prognostic factors on survival included incomplete surgical resection (*P*=0.001) and use of regimens without pre- and postoperative chemotherapy (*P*=0.007). Nine of the 25 patients were treated with pre- and postoperative chemotherapy and complete surgical resection. Among them, eight are alive with no disease. Radical surgical resection is the overriding prognostic factor for flat bone sarcomas in young patients. Nevertheless, our results suggest a more favourable outcome since the advent of intensive chemotherapy.

Malignant bone tumours are the sixth most common malignant neoplasms in children, accounting for approximately 5% of childhood malignancies (Young and Miller, 1975; Bernard *et al*, 1993). With the exception of Ewing's sarcoma, other primary flat bone sarcomas (osteosarcoma, malignant fibrous histiocytoma and chondrosarcoma) are rare in children and adolescents, and their incidence increases with advancing age (Boland and Huvos, 1986; Goorin *et al*, 1987; Huvos and Marcove, 1987; Tucker *et al*, 1987; Young *et al*, 1990). In contrast, bone sarcoma occurring as a second malignant neoplasm after irradiation is more likely to arise in flat bones of the axial skeleton than are *de novo* bone sarcomas (Weatherby *et al*, 1981; Huvos *et al*, 1985; Stea *et al*, 1987; Pratt *et al*, 1997; Tabone *et al*, 1999).

Advances in the treatment of primary osteogenic sarcoma of the extremities, and the demonstrated major impact of multiagent adjuvant chemotherapy in randomised trials, have markedly improved relapse-free survival (Link *et al*, 1986; Eilber *et al*, 1987; Saeter *et al*, 1991; Meyers *et al*, 1992; Meyer *et al*, 2001). However, the majority of studies generally excluded patients with osteosarcoma or other primary sarcomas of flat bones. Very limited information is available about the clinical behaviour and management of primary or radiation-related flat bone sarcomas in children or adolescents (Kellie *et al*, 1990; Kawai *et al*, 1998; Maes *et al*, 1998; Daw *et al*, 2000; Duffaud *et al*, 2000). Moreover, most of the published studies pooled pediatric and adult patients, and began before the era of intensive chemotherapy (Kellie *et al*, 1990; Daw *et al*, 2000). We described the outcome of pediatric and adolescent patients, on the one hand, because some studies suggested age as a prognostic factor (Raymond *et al*, 1987; Saeter *et al*, 1996), with a more favourable outcome in children and adolescents, and, on the other hand, because during the era of modern chemotherapy, pediatric and adult patients were treated according to different protocol.

To better define the natural history of these tumours and to propose management recommendations for children, we analysed the clinical characteristics, treatment modalities and outcome of 25 young patients with primary or radiation-related flat bone sarcoma (other than Ewing's sarcoma) treated in the Pediatrics Department at the Institut Gustave Roussy from 1981 to 1999. During this period, all patients were treated according to intensive chemotherapy: T10 protocol or modified T10 protocol (Kalifa *et al*, 1993).

## PATIENTS AND METHODS

From January 1981 to January 1999, 440 consecutive patients were admitted to the Pediatrics Department at the Institut Gustave Roussy for treatment of bone sarcomas other than Ewing's sarcoma. This group comprised children and adolescents diagnosed as having osteosarcoma (422), chondrosarcoma (14), malignant fibrous histiocytoma (3) and fibrosarcoma (1). Of these 440 patients, 29 were treated for flat bone sarcoma (representing 6.6% of all patients with non-Ewing's sarcoma of bone). Of these 29 patients, four were inevaluable because of treatment refusal and inadequate follow-up in one, and three were lost to follow-up. This retrospective analysis therefore concerns 25 children and adolescents. It reports on the outcome of patients as of April 2001, 15 months after the inclusion of the last case.

The diagnosis of flat bone sarcoma was based on conclusive clinical and imaging findings that were always confirmed by histological analysis. The initial work-up to determine the extent of the primary tumour and the presence of metastatic disease included computed tomography (CT) scan of the primary site (*n*=25) and chest (*n*=25), magnetic resonance imaging (MRI) of the primary site (*n*=20), conventional chest radiography (*n*=25) and technetium-99m-diphosphonate bone scan (*n*=25). All available histologic material was reviewed by one of us (JMG), who classified the tumours according to the World Health Organization (WHO) histologic typing of bone tumours (Contesso *et al*, 1987; Forest, 1995).

Response to preoperative chemotherapy, based on conclusive clinical and imaging findings (conventional radiography and CT scan of the primary site and metastases, skeletal scintigraphy and MRI of the primary site), was evaluated before surgical excision of the primary tumour. The overriding response criteria were, however, histopathologic findings after surgery. The surgical margin was defined as the closest margin between the tumour and the dissection plane. It was identified based on the description of the Musculoskeletal Tumor Society (Enneking, 1986). Surgical procedures were defined as a biopsy when an incisional biopsy was performed, as incomplete resection when tumour invasion of surgical margins was proven histologically, and as complete resection when tumour-free surgical margins were histologically confirmed. Response to preoperative chemotherapy was assessed in resected tumours by histologically grading the degree of tumour necrosis in the surgical specimen according to the method proposed by Huvos *et al* (Huvos *et al*, 1977; Rosen *et al*, 1982).

Follow-up was calculated from the time of diagnosis to the last contact. Actuarial survival curves were plotted from the diagnosis of flat bone sarcoma using the Kaplan–Meir method (Kaplan and Meier, 1958; Meyer *et al*, 1992). Survival distribution comparisons were performed using the logrank test. Analysis of the variables influencing survival was performed using a Cox proportional hazards model (Dixon, 1988).

## RESULTS

### Patient characteristics

A total of 25 children and adolescents (15 males and 10 females) treated in the Pediatrics Department were included in this retrospective study. Patient characteristics are listed in Table 1

**Table 1:** Patient characteristics and outcome

						**Treatment**				
**Patient no.**	**Sex**	**Age at diagnosis (year)**	**Primary site**	**Metastases**	**Histologic type**	**Chemotherapy**	**Surgery**	**Radiotherapy**	**Pattern of failure**	**Outcome/follow-up**
*Pelvis*										
1*	F	20	SACRUM	No	OST (F)	Pre- and postoperative	Incomplete resection	55 Gy	Local+metastatic	DOD/17 months
2*	F	10	ISCHIUM	No	OST	Pre- and postoperative	Resection	No		NED/3.5 years
3	M	16	ILIUM	No	OST (C)	Postoperative	Incomplete resection	No	Local	DOD/2.5 years
4	M	15	ILIUM	No	OST (C)	Palliative	No	16 Gy (neutron)		DOD/4 months
5	F	15	ILIUM	Yes	CHON	Yes	No	40 Gy (palliative)		DOD/11 months
6	M	16	ILIUM	No	CHON	Pre- and postoperative	Resection	No		NED/6.5 years
7	M	15	ISCHIUM	No	OST (C)	Pre- and postoperative	Resection	25 Gy (neutron)	Local +metastatic	DOD/3.5 years
								35 Gy (palliative)		
8	M	17	ILIUM	No	CHON	Preoperative	Resection	No		NED/4 years
9	F	13	ILIUM	No	OST (O)	Pre- and postoperative	Resection	No		NED/3 years
10	F	16	SACRUM	No	OST	Yes	No	40 Gy	Local	PD/2.5 years
								30 Gy (neutron)		
11*	M	15	ILIUM	No	OST	Pre- and Postoperative	Resection	No	Local	NED/11.5 years
										
*Craniofacial*										
12*	M	23	MANDIBLE	No	OST	Pre- and postoperative	Hemimandibulectomy	No		NED/5.5 years
13*	M	15	OCCIPUT	No	OST (M)	Pre- and postoperative	Incomplete resection	No	Local	DOD/2 years
14	F	9	MANDIBLE	Yes	OST (M)	Preoperative	Hemimandibulectomy	No	Local	DOD/6 months
15	M	16	MANDIBLE	Yes	OST	Pre- and postoperative	Hemimandibulectomy	55 Gy	Local + metastatic	NED/8.5 years
16*	M	18	MANDIBLE	No	OST (M)	Preoperative	Hemimandibulectomy	No	Local +metastatic	DOD/7 months
17*	M	17	MAXILLA	No	OST	Yes	No	No		DOD/11 months
18*	F	7	ZYGOMA	No	OST	Yes	No	No		DOD/8 months
19	F	12	MAXILLA	No	OST	No	Incomplete resection	56 Gy	Local	DOD/3 years
20	F	2	MAXILLA	No	MFH	Pre- and postoperative	Zygoma and maxilla Incomplete resection	No		NED/5 years
										
*Other sites*										
21	M	17	VERTEBRA	No	OST (C)	Postoperative	Incomplete resection	22 Gys (telecobalt)	Local	DOD/2 years
								50 Gys		
22	F	4	VERTEBRA	No	CHON	Postoperative	Incomplete resection	45 Gys	Local	DOD/4.5 years
								22 Gys (palliative)		
23	M	12	VERTEBRA	Yes	OST (m)	Yes (HDC)	No	40 Gys		NED/8 years
24*	M	17	CLAVICLE	No	OST (M)	Pre- and postoperative	Cleidectomy	No		NED/9 years
25*	M	15	SCAPULA	No	OST (C)	Pre- and postoperative	Scapulectomy	No		NED/11.5 years

* Radiation-induced osteosarcoma; OST=osteosarcoma; CHON=chondrosarcoma; MFH=malignant fibrous histiocytoma; F=fibroblastic; C=chondroblastic; O=osteoblastic; M=mixed; m=microcellular; DOD=died of disease; NED=no evidence of disease; PD=progressive disease.

. The median age was 15 years (range 2–23). Nine tumours were located in the craniofacial bones, 11 in the pelvis and five in flat bones at other sites. When more than one bone was involved, the bone predominantly involved was considered as the primary site. The histological types in this group of children and adolescents were osteosarcoma (20), chondrosarcoma (four) and malignant fibrous histiocytoma (one). At diagnosis, four patients (16%) presented with metastases in the lungs only (two), in the lungs and bones (one) and in the lungs and subcutaneous tissue (one). Secondary flat bone osteosarcoma within a previously irradiated site was diagnosed in 10 cases (40%).

### Radiation-related osteosarcoma

All postradiation osteosarcoma developed within the radiation field. The type of primary tumour is listed in Table 2

**Table 2:** Radiation-related flat bone sarcomas

		**Therapy for primary tumour**		
**Patient no.**	**Primary tumour**	**Chemotherapy**	**RT (Gy)**	**Time to onset (year)**
1	Bone giant cell tumour	No	15	7.1
2	Nephroblastoma	Actino, Doxo	30	3.3
12	Rhabdomyosarcoma	VCR, Actino, Doxo	45	7.8
13	Rhabdomyosarcoma	VCR, Actino, Doxo, Cyclo	45	13.3
14	Medulloblastoma	Etoposide, Cyclo, Carbo	55	6.7
17	Rhabdomyosarcoma	VCR, Actino, Cyclo, Procarb	45	15.5
18	Undifferentiated carcinoma of the nasopharyngeal type (UCNT)	No	65	14
19	Neuroblastoma	VCR, Doxo, Cyclo	30	6.3
26	Hodgkin's disease	VCR, PDN, Procarb, Chlormeth	40	11.8
28	Hodgkin's disease	VCR, PDN, Procarb, Chlormeth	40	9.8

VCR=vincristine; Actino=actinomycin; Doxo=doxorubicin; Cyclo=cyclophosphamide; Carbo=carboplatin; Procarb=procarbazine; PDN=prednisone; Chlormeth=chlormethin; UCNT=undifferentiated carcinoma of the nasopharyngea type.

. The median time between radiotherapy and the diagnosis of the secondary bone sarcoma was 7 years (range 3–15). The sites of postirradiation tumours were the craniofacial bones (five), the pelvis (three), the clavicle (one) and the scapula (one). Distant metastases were not identified at diagnosis in any of these 10 patients. The median dose of radiation administered within the target volume where the secondary bone sarcoma had developed was 40 Gy (range 15–65). Eight of these 10 patients had also received prior therapy with alkylating agents (Table 2).

### Histopathologic findings

Upon review, 19 of the 25 tumours were classified as osteosarcomas (Table 1). The slide of the tumour in one additional patient was not available for central review. Notwithstanding, the microscopic description recorded on the pathology report of this patient corresponded to the diagnosis of osteosarcoma. Histologic subtyping was available for 12 of 19 flat bone osteosarcomas. The predominant histologic subtypes in these 12 tumours were chondroblastic (five cases), osteoblastic (one), fibroblastic (one) and mixed chondroblastic and osteoblastic (four). The last patient (patient 23) had a small cell osteosarcoma. Concerning the seven patients for whom histologic subtyping was not available, five had a craniofacial tumour and two a pelvis tumour.

Four patients in this series had chondrosarcoma that were poorly differentiated in two cases and well differentiated in two. The last patient (patient 21) had a typical malignant fibrous histiocytoma.

### Therapy and outcome

The treatment of patients with flat bone sarcoma is listed in Table 1. One patient (no. 4) was exclusively treated with a palliative intent. He had a large tumour involving the pelvis and thigh, which prohibited surgical excision. He died of disease progression 4 months after the diagnosis.

The 24 remaining patients were treated with a curative intent. One patient (no. 19), who underwent incomplete surgical resection followed by radiotherapy, relapsed 3 years later and died. Intensive chemotherapy regimens were administered to 23 patients, three of whom (nos. 3, 21, 22) only received postoperative chemotherapy. Surgical resection had been incomplete in these three patients who received regimens containing high-dose methotrexate (HDMTX) postoperatively. All the three died of disease. The 20 remaining patients received chemotherapy as first-line treatment. Only chemotherapy and radiotherapy were used to treat the primary tumour in five of them (nos. 5, 10, 17, 18, 23), with HDMTX in three out of five. Surgical excision of the primary tumour was impossible in four of the five patients due to early progression, despite second-line therapy with cisplatin, ifosfamide and doxorubicin. Three of them died of disease (11, 11 and 8 months post diagnosis). The fourth patient (no. 10) is alive with progressive disease 2.5 years after diagnosis. The last patient (no. 23), who had small-cell osteosarcoma of the first vertebra, was treated according to the protocol for metastatic Ewing's sarcoma (Sandoval *et al*, 1996). Having achieved a complete remission at all metastatic sites on first-line chemotherapy, he was consolidated with high-dose chemotherapy (HDC) followed by stem cell transplantation (SCT) and irradiation to the primary site. He is alive and free of disease with a follow-up of 8 years. Three patients (nos. 8, 14, 16) were treated with preoperative chemotherapy and surgery: one of them (no. 8) had a complete surgical resection after one course of HDMTX (12 g m^−2^), and is alive and free of disease with a follow-up of 4 years. In the two others (nos. 14, 16), resection of the primary was incomplete after one and three courses of chemotherapy, respectively. They both relapsed rapidly and died of disease 6 and 7 months after the diagnosis.

The 12 remaining patients received chemotherapy before and after surgical resection. Of them, 11 received regimens containing high-dose methotrexate (HDMTX; 8–12 g m^−2^) according to the modified T10 protocol (Rosen *et al*, 1982) with two to eight (median 7) preoperative HDMTX courses as first-line chemotherapy. The 12th patient (no. 20), who had malignant fibrous histiocytoma, was treated with a combination of ifosfamide, vincristine and actinomycin in three preoperative courses, but failed to achieve a measurable response. He received three courses of doxorubicin and cisplatin, and two courses of HDMTX as second-line chemotherapy, but again no response was observed.

Of these 12 patients, 11 were assessable for a histologic response to preoperative chemotherapy. The postoperative results and details are listed in Table 3

**Table 3:** Results and postoperative treatment according to preoperative chemotherapy

**Preoperative chemotherapy**	**Histologic response ⩽5% of viable cells**	**Postoperative chemotherapy (No patient)**
HDMTX and doxorubicin-containing regimens	0/3	Ifosfamide and cisplatin-containing courses (1, 24, 25)
HDMTX and ifosfamide-containing regimens	5/8	HDMTX and ifosfamide-containing courses for good responders (2, 7, 11, 12, 13)
		Ifosfamide and cisplatinum for poor responders (6, 9, 15)

. Surgical resection was microscopically complete in eight of the 12 patients. Seven of them (nos. 2, 6, 9, 11, 12, 24 and 25) are alive and free of disease, with a follow-up of 3–11.5 years, and one (no. 7) relapsed 31 months post diagnosis and died. Among the four patients (nos. 1, 13, 15 and 20) in whom surgery was incomplete, two received postoperative radiotherapy to the site of the primary tumour (nos. 1, 15). Two of these four patients died of disease and two are alive with no evidence of disease, with a follow-up of 5 and 8.5 years, respectively (nos. 15, 20).

### Prognostic factors

**Figure 1:**
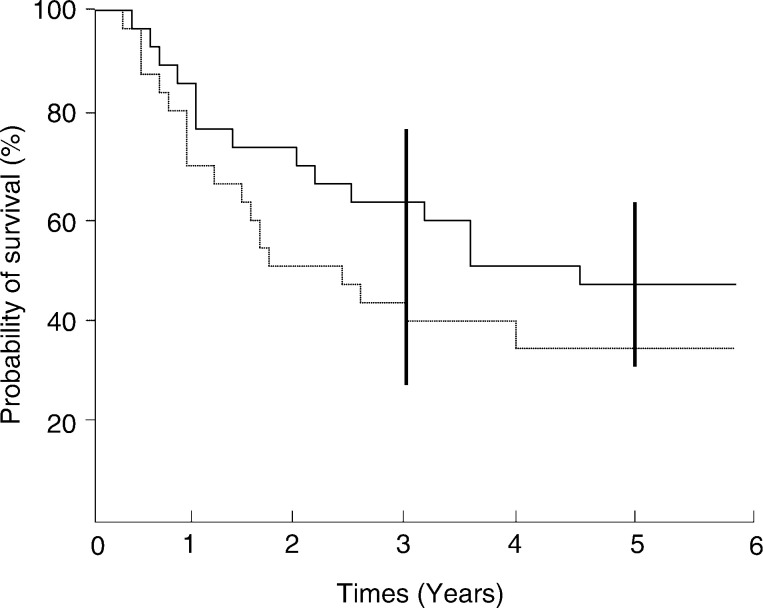
Survival rates from the diagnosis of flat bone arcomas: ______ Overall survival (*n*=25); ....event-free survival (*n*=25).

The outcomes of the 25 patients with flat bone sarcomas are shown in Figure 1

. As of April 2001, 11 out of 25 patients were alive and disease-free, with a median follow-up of 5.3 years post diagnosis (range 3–11.5). The projected overall survival (OS) and event-free survival (EFS) rates at 5 years were 45.1% (±19%) and 34.3% (±18%) for all 25 patients with flat bone sarcomas (Figure 1). The factors likely to influence EFS were studied by univariate analysis (logrank test). The EFS rate of patients with a secondary bone sarcoma was similar to that of patients with *de novo* flat bone sarcomas (*P*=0.1). Sex, age at diagnosis, the site of the primary as well as detectable metastases at diagnosis had no significant impact on outcome. With regard to the histologic subtype, eight of the 20 patients with osteosarcoma and two of the four patients with chondrosarcoma survived. The number of cases per histologic subtype is, however, too small to allow any statistical comparison. Of the 12 patients who received preoperative chemotherapy, 11 were assessable for a histologic response. Surprisingly, three of the five patients with tumour necrosis ⩾95% and five of the six patients with tumour necrosis <95% survived. Concerning chemotherapy, the comparison of EFS between patients with or without pre- and postoperative chemotherapy indicated significantly poorer survival in the subgroup without chemotherapy (*P*=0.007). However, the most powerful prognostic indicator was incomplete surgical resection: indeed, only two of the 10 patients in whom tumour resection was incomplete survived. Conversely, eight of the nine patients in whom surgery was complete survived. EFS was significantly higher in these nine patients (71.1% (±5.5%)/12.5% (±10.6%): *P*=0.001).

## DISCUSSION

We reviewed our institutional experience with flat bone sarcomas, other than Ewing's, in order to characterise the clinical findings and to identify factors that have an impact on outcome. To our knowledge, this is the first study to focus on pediatric primary and secondary flat bone sarcomas, during the era of intensive chemotherapy (Kellie *et al*, 1990; Kawai *et al*, 1998; Maes *et al*, 1998; Daw *et al*, 2000; Duffaud *et al*, 2000). Our patient characteristics were comparable to those of other published studies (Kellie *et al*, 1990; Kawai *et al*, 1998; Maes *et al*, 1998; Daw *et al*, 2000; Duffaud *et al*, 2000). The median age was 15 years (range 2–23), confirming that flat bone sarcoma affects older children more frequently than conventional extremity osteosarcoma (Whelan, 1997).

Only four patients in this series had chondrosarcoma, three of which arose in the pelvis and two had high-grade chondrosarcoma. Our results confirm the poor chemosensitivity and radiosensitivity of these tumours (Lee *et al*, 1999; Bergh *et al*, 2001): one of the two patients with high-grade chondrosarcoma had a measurable response after chemotherapy and the only patient in whom radiotherapy was delivered with a curative intent, relapsed 13 months later and died of disease. The most powerful prognostic factor concerning flat bone chondrosarcomas appeared to be the quality of the surgical resection. Among the four patients with chondrosarcoma, two are alive and disease free and surgical resection was complete in both of them, whereas the two other patients died of disease after incomplete surgical resection.

Some studies have already indicated that flat bone osteosarcomas often respond poorly to chemotherapy and progress in their treatment has not improved to the same extent as that accomplished for long bone osteosarcomas (Link *et al*, 1986; Eilber *et al*, 1987; Saeter *et al*, 1991; Meyers *et al*, 1992). The chondroblastic variant has been reported to be somewhat over-represented among flat bone osteosarcomas. Kellie *et al* (1990) reported that 37% of 19 flat bone osteosarcomas were predominantly chondroblastic. In our study, 20% of the osteosarcomas were predominantly chondroblastic. Chondroblastic variants of osteosarcoma, such as chondrosarcoma, respond poorly to chemotherapy, with 3–6.1% of total necrosis after initial chemotherapy (Ozaki *et al*, 1997; Bacci *et al*, 1998; Lee *et al*, 1999; Bacci *et al*, 2001; Bergh *et al*, 2001), whereas 39–52% of extremity osteosarcoma had >95% of tumour necrosis following preoperative chemotherapy (Sandoval *et al*, 1996; Bacci *et al*, 1998, 2001). The excess incidence of the chondroblastic variant could then explain the poor chemosensitivity of flat bone osteosarcomas. Moreover, comparison of flat bone osteosarcoma and extremity osteosarcoma revealed that osteosarcomas of flat bones had the worst prognosis.

Evidence reported on the prognosis of secondary osteosarcomas compared to primary lesions is inconsistent and controversial. In a recent study, secondary osteosarcomas of the pelvis were found to be correlated with a poor prognosis (Grimer *et al*, 1999). In contrast, Pratt *et al* (1997) published that the prognosis and overall outcome of patients with flat bone tumours were comparable for primary and secondary osteosarcomas. In another study, Tabone *et al* (1999) examined 23 cases of radiation-related osteosarcoma after treatment of childhood cancer. In all, 12 out of 23 patients were alive with no evidence of disease, and six out of 14 patients with flat bone osteosarcomas compared to three out of eight in our study. These results were confirmed by another study (Bielack *et al*, 1999). The present study appears to confirm that EFS of patients with secondary bone sarcoma is similar to that of patients with *de novo* flat bone sarcomas, and thus confirms that incomplete surgical resection of the primary tumour is the worst prognostic factor for flat bone osteosarcomas.

One of the aims of this study was to examine the prognostic factors usually analysed in flat bone sarcomas. The overall survival rate at 5 years is 45.1% (±19%) for the study population. Other studies have reported similar 5-year survival rates of between 33 and 47% for patients with pelvic osteosarcoma (Kawai *et al*, 1998; Grimer *et al*, 1999). In our study, the site of the tumour in the pelvis had no impact on outcome. Kellie *et al* (1990) reported a lower 10-year survival rate of 24%. However, their study focused on the outcome of patients treated between 1962 and 1987, before the era of intensive chemotherapy.

In contrast to other reported studies in which none of the patients had metastatic disease at presentation (Kellie *et al*, 1990), distant metastases were identified in four patients at diagnosis in our study. Metastases at diagnosis appeared to have no impact on outcome, but the number of cases was too small to allow any statistical comparison.

The efficacy of multidrug chemotherapy for patients with primary osteosarcoma of long bones amenable to *en bloc* resection is well documented (Huvos *et al*, 1985; Link *et al*, 1986; Eilber *et al*, 1987; Saeter *et al*, 1991; Meyers *et al*, 1992; Meyer *et al*, 2001). Kellie *et al* (1990) advocate the use of preoperative multiagent chemotherapy to facilitate surgical resection of non-Ewing's sarcomas of flat bone. Duffaud *et al* (2000) reported no survivors among eight out of 16 patients who developed metastases, and then recommended the use of pre- and postoperative chemotherapy. In our study, patients who received pre- and postoperative chemotherapy had a significantly better outcome than those who received another treatment modality (none, neoadjuvant or adjuvant chemotherapy).

The importance of complete resection in maximising the chances of prolonged relapse-free survival is supported by many authors (Kellie *et al*, 1990; Kawai *et al*, 1998; Grimer *et al*, 1999; Daw *et al*, 2000; Duffaud *et al*, 2000; Ferguson *et al*, 2001; Ozaki *et al*, 2002). Kawai *et al* (1998) identified two prognostic factors in a multivariate analysis of pelvic bone sarcomas: the type of surgical resection and the surgical margin. Daw *et al* (2000) reported that incomplete resection of osteosarcoma was associated with local failure and poor outcome in sarcomas of the head and neck. More recently, Gadwal *et al* (2001) reported a 5-year survival rate of 72% for 15 children who underwent surgical resection of primary osteosarcoma of the head and neck. In the largest study of sarcomas of flat bones in children and adolescents, Kellie *et al* (1990) reported complete surgical excision in 10 out of 28 patients, eight of whom also received postoperative chemotherapy. Five of these patients are still disease free. Surgical options for local control of flat bone osteosarcomas are more limited than for long bone lesions. Radical surgical resection is often impossible in critical areas, such as vertebrae, the sacrum or craniofacial bones, because there is a high risk of functional incapacitation or cosmetic defects. Concerning sarcoma of the pelvic bones, Kawai *et al* (1998) emphasised how it is often difficult to resect such lesions with wide margins, because of poor compartmentalisation and the complicated anatomy of the pelvis. Our results confirm that complete surgical excision is the strongest prognostic factor concerning flat bone sarcomas. In our study, 19 patients had surgical resection of the primary tumour (in six patients, surgical resection was impossible). Eight of nine patients who underwent complete surgical excision were alive with no evidence of disease, compared to two of 10 in whom resection of the primary tumour was incomplete. After complete surgical resection, local recurrence occurred in 22% of cases. The number of patients is, however, too small to suggest that local recurrence has an effect on survival. Adequate local control was achieved in five of the 10 patients in whom resection was incomplete with additional, external beam, high-energy RT (Smith, 1982). Local control was prolonged in these five patients, but only one is still alive with no evidence of disease, having attained a follow-up of 8.5 years. It is difficult to assess the role of postoperative radiotherapy, given the heterogeneity of each small series.

In conclusion, this study has confirmed that radical surgical resection is the overriding prognostic factor for flat bone sarcomas in children and adolescents. Nevertheless, our results suggest a more favourable outcome since the advent of intensive chemotherapy. Usually, patients with osteosarcoma of flat bones are excluded from clinical trials of new adjuvant therapies. Given the poor response to conventional chemotherapy, new therapeutic approaches are warranted. We propose the conduct of an international prospective study with a large population, to evaluate the role of each treatment modality and to study the specific biology of these tumours.
